# Knowledge domains and emerging trends in non-invasive brain stimulation: A bibliometric analysis via CiteSpace

**DOI:** 10.7705/biomedica.7040

**Published:** 2024-05-31

**Authors:** Inmaculada Ruiz-López, Desirée Victoria-Montesinos, Juan Martínez-Fuentes

**Affiliations:** 1 Programa de Doctorado en Ciencias de la Salud, Universidad Católica de Murcia, Murcia, España Universidad Católica de Murcia Universidad Católica de Murcia Murcia España; 2 Facultad de Farmacia y Nutrición, Universidad Católica de Murcia, Murcia, España Universidad Católica de Murcia Universidad Católica de Murcia Murcia España; 3 Facultad de Fisioterapia, Podología y Terapia Ocupacional, Universidad Católica de Murcia, Murcia, España Universidad Católica de Murcia Universidad Católica de Murcia Murcia España

**Keywords:** transcranial magnetic stimulation, bibliometrics., estimulación magnética transcraneal, bibliometría.

## Abstract

**Introduction.:**

In recent decades, new non-invasive brain stimulation techniques and protocols have been developed, such as transcranial magnetic stimulation and transcranial direct current stimulation.

**Objective.:**

To identify and visualize the intellectual structure of non-invasive brain stimulation through document co-citation analysis.

**Materials and methods.:**

We analyzed 30,854 Web of Science-indexed manuscripts and their 1,615,692 references regarding non-invasive brain stimulation, all published from 1988 to 2022. We drew a document co-citation network map using CiteSpace software.

**Results.:**

The most productive journal was *Clinical Neurophysiology*. The most published institution was the University College London, and the country with the most reports was the USA. The most productive author was Alvaro Pascual-Leone and the most cited author in the non-invasive brain stimulation field was J. C. Rothwell. In addition, the most cited study was that of Rossi *et al*. (2009). The safe application of non-invasive brain stimulation techniques and their effects on motor or executive functions is an emerging trend in this research area.

**Conclusions.:**

The current investigation displayed a quantitative scientometric approach and delved into the advancement of non-invasive brain stimulation research by examining the references published in this domain. These findings can be valuable for professionals to picture the patterns of recognition and emerging directions in the field.

## Resumen

**Introducción.:**

En las últimas décadas se han desarrollado nuevas técnicas y protocolos de estimulación cerebral no invasiva, como la estimulación magnética transcraneal y la estimulación transcraneal con corriente directa.

**Objetivo.:**

Identificar y visualizar la estructura intelectual de la estimulación cerebral no invasiva a través del análisis de cocitación de documentos.

**Materiales y métodos.:**

En este estudio se analizaron 30.854 manuscritos indexados en Web of Science sobre estimulación cerebral no invasiva, todos ellos publicados entre el 1988 y el 2022, y sus 1.615.692 referencias. Se dibujó un mapa de red de cocitación de documentos utilizando el software CiteSpace.

**Resultados.:**

La revista más productiva fue *Clinical Neurophysiology*. La institución y el país con más reportes fueron *University College London* y Estados Unidos. El autor con más publicaciones fue Álvaro Pascual-Leone y el autor más cocitado en el campo de estimulación cerebral no invasiva fue J. C. Rothwell. Además, el estudio más citado fue el de Rossi *et al*. (2009). La aplicación segura de las técnicas de estimulación cerebral no invasiva y sus efectos sobre las funciones motoras o ejecutivas es una tendencia emergente de la investigación en esta área.

**Conclusiones.:**

La presente investigación muestra un enfoque cienciométrico cuantitativo y profundiza en el avance de la investigación sobre la estimulación cerebral no invasiva mediante el examen de las referencias publicadas en este ámbito. Estos resultados pueden ser de gran utilidad para los profesionales que deseen hacerse una idea de los patrones de reconocimiento y las nuevas orientaciones en este campo.

Non-invasive brain stimulation (NIBS) has attracted the interest of the general public and researchers since Anthony Barker first described transcranial magnetic stimulation at the University of Sheffield (UK) in 1985. Consequently, there has been an increasing number of scientific publications in this field ^1^^-^^3^. In recent decades, new NIBS techniques and protocols have been developed, including transcranial magnetic stimulation (TMS) and transcranial direct current stimulation (tDCS) ^4^^,^^5^.

For the development of these techniques, as in other scientific areas, researchers are obliged to consult large amounts of scientific literature to develop their work, which involves a high time expenditure and complexity ^6^^,^^7^. Thus, as we move towards an information and knowledge society, it is necessary to have quantitative indicators and tools that make it possible to objectify the differences between the publications ^6^^,^^8^.

For several decades, methodological models have been developed that allow us to understand the development of scientific activity. Bibliometric studies offer a statistical and quantitative analysis of published articles and provide insight into their impact on a field of research ^9^^-^^13^.

The first works on bibliometrics were carried out by Garfield, Kessler, and Price ^14^^-^^16^, who observed that in the statistical analysis of bibliographic references and citations, could be found patterns establishing thematic associations between scientific works ^9^^,^^14^^-^^18^. Years later, Small and Marshakova ^19^^,^^20^ proposed co-citation analysis as an objective model to reveal the intellectual structure of scientific specialties ^9^^,^^19^^,^^20^.

Co-citation analysis is based on the hypothesis that there is a thematic similarity between two or more documents cited in the same document, and the higher the co-citation frequency, the greater the affinity between them ^9^. If a co-citation analysis is performed correctly, it will be possible to know the most relevant authors or papers in a discipline through the empirical consensus established by the hundreds who cited those authors or papers and not only by the impressions of a single researcher ^6^^,^^17^^,^^21^.

Most cited papers represent the key concepts, methods, or experiments in a field, so co-citation patterns can be used as a technique to contribute to the knowledge of the scientific disciplines intellectual structure ^9^^,^^22^. Bibliometric studies apply to areas like neurology where similar analyses have been performed for other neuropsychiatric treatments ^23^^-^^26^.

On the other hand, CiteSpace is a freely available Java software invented in 2004 by Professor Chaomei Chen to perform bibliometric analysis. It is characterized by analyzing and visualizing network maps of authors, keywords, institutions, countries, subject categories, and co-citation networks of cited authors, cited references, and cited journals ^11^^,^^27^^-^^33^.

The graphs obtained from CiteSpace are composed of two main elements: the nodes and the links ^6^^,^^11^^,^^31^. Each node represents elements such as citation, institution, author, and country, and each link between two nodes involves a co-citation relationship. Thus, the size of the nodes represents the individual citation frequency of each document, and the thickness of the links represents the co-citation strength between two nodes. Additionally, the grey tone of the nodes and lines represents different years ^6^^,^^11^^,^^31^.

After what was exposed, the main objective of this study is to identify and visualize the intellectual structure of non-invasive brain stimulation through document co-citation analysis.

## Material and methods

The data utilized for bibliometric analysis was sourced from the Web of Science Core Collection by Clarivate Analytics ^26^. The index term included ‘“non-invasive brain stimulation’ OR ‘non-invasive electrical brain stimulation’ OR ‘non-invasive magnetic brain stimulation’ OR ‘transcranial direct current stimulation’ OR ‘transcranial magnetic stimulation’”. As a result, 30,854 studies were identified, encompassing 25,993 research originals and reviews, with a cumulative count of 1,615,692 references. These searched records were exported to CiteSpace for further analysis. The studies were downloaded on March 24, 2022. Each download study included full records and cited references. Inclusion criteria were original articles and reviews on non-invasive brain stimulation retrieved from the Web of Science published from 1985 to 2022. No exclusion criteria were described.

CiteSpace is a Java-based software utilized for the visualization of scientific bibliometric analysis ^28^. For this study, the chosen timeframe comprehended from January 1988 to December 2022, using a time slice of 5 years. Selection criteria were the top 50 items more cited per slice and the rest of the settings as default ^26^^,^^27^.

We observed the number of publications on NIBS each year, then studied and performed an analysis of the most productive journals and authors as well as the most co-cited authors, institutions, countries, and documents. Finally, we used three labeling algorithms to find out the topics analyzed in the studies of each cluster: Latent Semantic Indexing (LSI), Log-likelihood ratio (LLR), and mutual information (MI), and we analyzed the burst citations to identify emerging trends ^26^^,^^27^.

The indicators used were the number of citations received, centrality, and the strongest citation bursts. The network maps obtained from the CiteSpace software are made of nodes and links. The size of the nodes represents the number of citations received by an item and the thickness of the links; the short distance between two nodes represents the co-occurrence strength between two items.

This bibliometric study uses secondary databases in the public domain and does not require the approval of an institutional ethics committee.

## Results

### Publication years and journals

**Figure 1.**
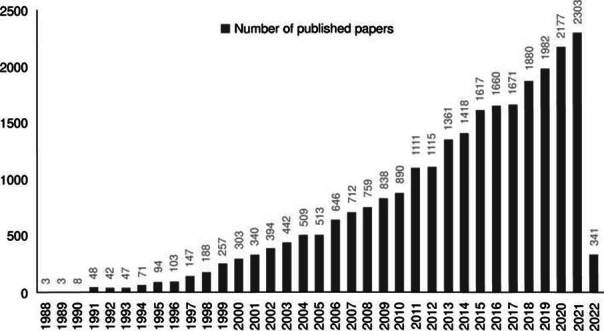
Number of papers on non-invasive brain stimulation published from 1988 to 2022.

As shown in figure 1, the total number of publications increased from 1988 to 2022. The examined timeframe was categorized into three distinct stages: the initial stage spanning from 1988 to 1995, the second stage encompassing the years from 1996 to 2010, and the third stage comprising from 2011 to 2022. The first period is characterized by the rapid growth of the publications’ number (from 3 publications in 1988 to 94 publications in 1995). The period from 1996 to 2010 had a progressive development, while the third period showed an explosive growth because the total number of publications (18,636) was higher than that from the two previous periods combined (7,357).

The top ten journals with the highest volume of published research on NIBS are in table 1, serving as a valuable point of reference for new researchers. NIBS articles are distributed in a total of 2,310 journals. The most productive journal was Clinical Neurophysiology (928 articles), and the second-ranked was Brain Stimulation (854 articles).

**Table 1.** Top 10 most productive journals

Journals	Number of published papers	Impact factor
Clinical Neurophysiology	928	4.861
Brain Stimulation	854	9.184
Experimental Brain Research	745	2.064
Frontiers in Human Neuroscience	567	3.473
Neuroimage	510	7.400
Plos One	488	3.752
Neuroscience Letters	450	3.197
Journal of Neurophysiology	435	2.974
Journal of Neuroscience	426	6.709
Neuropsychologia	402	3.054

### Author and co-authorship

Knowledge maps can offer insights into prominent authors and assist researchers in forging collaborative connections. Table 2 shows the top 10 authors who have published articles related to NIBS. The most productive author was Álvaro Pascual-Leone.

**Table 2.** Top 10 active authors

Journals	Number of published papers
Pascual-Leone A	478
Rothwell JC	390
Fregni F	375
Daskalakis ZJ	304
Fitzgerald PB	288
Paulus W	283
Nitsche MA	278
Hallett M	248
Ziemann U	239
Cohen LG	219

**Figure 2.**
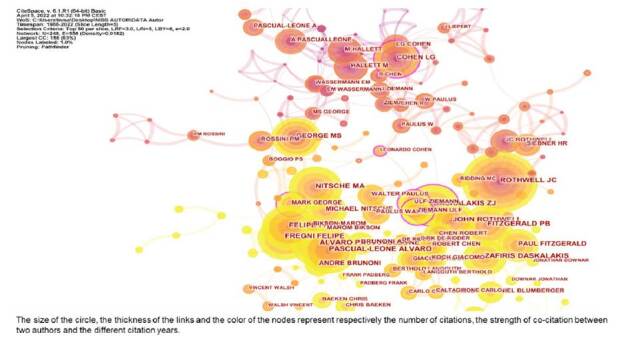
Co-authorship of non-invasive brain stimulation research

Figure 2 displays the co-authorship network; it contains 248 unique nodes and 558 links. The size of the circle represents the number of citations received by the author, and the link thickness represents the co-citation strength between the two authors. The color of the nodes represents different years. In figure 2, we observe that the most representative author in the field of NIBS was J. C. Rothwell with 390 citations, followed by Álvaro Pascual-Leone (352) and Felipe Fregni (337).

### Co-institute and co-country

Table 3 shows the top 10 institutes and countries that have published articles related to NIBS. We can highlight the most productive institutions have been the University of London (1,434 publications) and Harvard University (1,423 publications), while the countries with the most publications on NIBS are the United States of America (7,497) and Germany (4,012).

**Table 3.** Top 10 active institutions and countries in NIBS

	Institution			Countries	
Ranking	Institution	Number of oublished oaoers	Ranking	Country	Number of published oaoers
1	University of London	1,434	1	USA	7,497
2	Harvard University	1,423	2	Germany	4,012
3	University College London	1,157	3	Italy	3,424
4	University of Toronto	801	4	England	3,004
5	National Institutes of Health (NIH) - USA	775	5	Canada	2,242
6	Beth Israel Deaconess Medical Center	738	6	Australia	2,104
7	University of California System	702	7	China	1,565
8	Instituí National de la Sante et de la Recherche Medícale (INSERM)	632	8	Japan	1,546
9	NIH National Institute of Neurological Disorders Stroke (NINDS)	561	9	France	1,404
10	Centre National de la Recherche Scientifique (CNRS)	526	10	Netherlands	984

NIBS: Non-invasive brain stimulation

**Figure 3.**
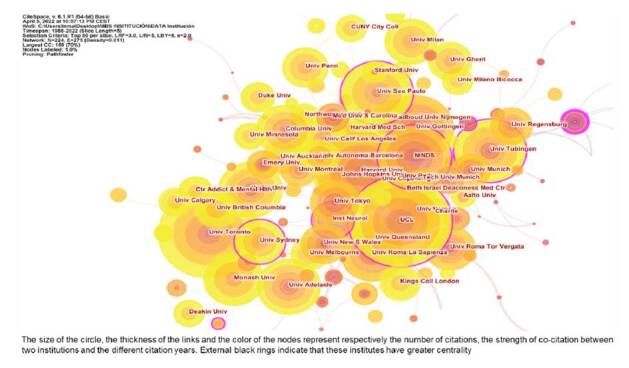
Co-institutes in the field of non-invasive brain stimulation

**Figure 4.**
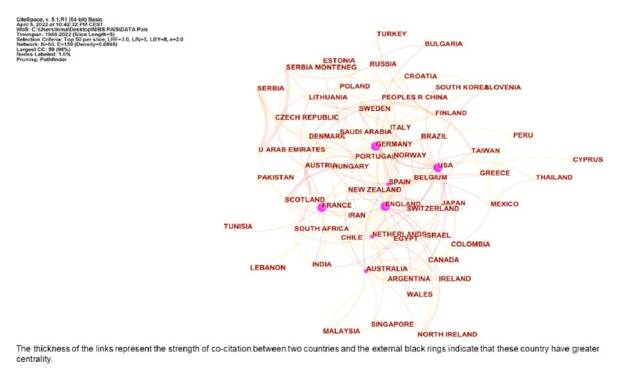
Co-countries in the field of non-invasive brain stimulation

Figure 3 displays co-institute results in the field of NIBS. The citation number per institute is represented by the size of the circle. The thickness of the links and the short distance between the two circles represent the co-occurrence strength between two institutes. The institution with the highest citation frequency was the University College London in the UK (764 citations), followed by Harvard University (720) and the University of Toronto (695). External black rings indicate that these institutes have greater centrality. The institutions with the highest centrality were the National Institute of Neurological Disorders Stroke - NINDS (0.21) in USA, followed by the University of Sidney (0.18) and the University College London (0.17). Figure 4 exhibits co-country results in the field of NIBS. The countries receiving the most citations are USA (7,428), Germany (3,946) and Italy (3,410). In addition, we can observe that the countries with the highest centrality were USA (0.40), England (0.33), and Germany (0.26).

### Document co-citation analysis

**Figure 5.**
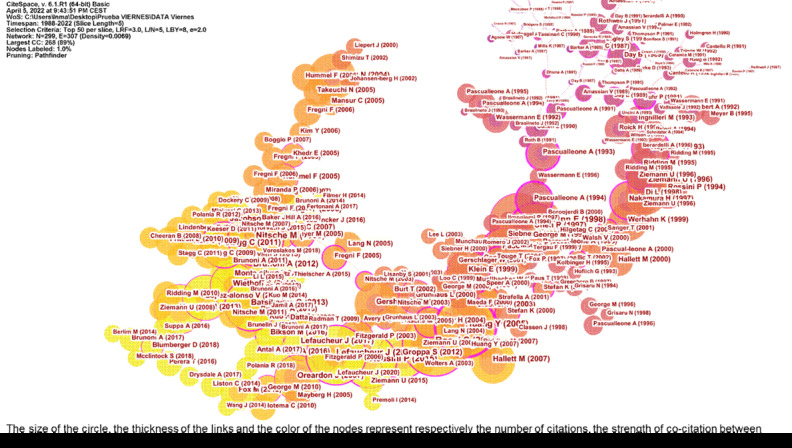
Document co-citation analysis in non-invasive brain stimulation research

We analyzed 25,993 studies using the CiteSpace software. A map of the document co-citation network is shown in figure 5 and contains 299 nodes and 307 lines. These nodes and lines represent the number of citations each study has received and the co-citations relationship of the collected studies, respectively. The node size increases with higher citation counts for the study, while the color and thickness of the circle within the node reflect the citation frequency across various periods. Internal rings represent earlier cited studies, while external rings represent more recently cited studies. The width of an annual ring corresponds to the number of citations within a specific period.

The most cited papers are Rossi *et al.*^34^ in cluster 11 with a total of 1,082 citations, followed by Rossini *et al*.^35^ in cluster 11 with 610 citations, Huang *et al*. ^36^ in cluster 6 with 537 citations, and Nitsche *et al*. ^37^ in cluster 10 with 527 citations.

The co-citation analysis of NIBS papers generated 17 co-citation clusters, each labeled with Indexed terms derived from their citations. To find out the topics analyzed in the studies of each cluster, CiteSpace can extract noun phrases from article titles for clustering based on three labeling algorithms: Latent Semantic Indexing (LSI), Log-likelihood ratio (LLR), and mutual information (MI). The log-likelihood ratio typically yields superior outcomes regarding the distinctiveness and scope of topics linked to clustering ^27^. Table 4 presents an overview of the 17 clusters, with a contour value exceeding 0.8, indicating dependable and significant results.

**Table 4.** The 17 clusters of non-invasive brain stimulation document co-citation, identified by subject headings

Cluster ID	Size	Silhouette	Mean (cite year)	LSI	LLR	Label MI
0	22	0.908	1994	Motor cortex	Single motor unit	Cervical nerve root compression
1	21	0.966	2018	Cortical excitability	Motor learning	Cervical nerve root compression
2	20	0.887	2003	Human motor cortex	Human motor cortex	Cervical nerve root compression
3	19	0.986	1997	Silent period	Silent period	Rapid finger movement
4	18	0.916	2005	Major depression	Electroconvulsive therapy	Cervical nerve root compression
5	18	1	2018	Treatment-resistant depression	Treatment-resistant depression	Cervical nerve root compression
6	18	1	2009	Human motor cortex	Human motor cortex	Cervical nerve root compression
7	17	0.974	2019	Working memory	Prefrontal transcranial direct current stimulation	Cervical nerve root compression
8	16	0.9	1992	Motor evoked-potential	Motor evoked-potential	Human motor cortex
9	16	0.891	2002	Human motor cortex	Intracortical inhibition	Cervical nerve root compression
10	15	0.982	2012	Psychiatric disorder	Current density	Cervical nerve root compression
11	15	0.91	2018	Cortical excitability	Human motor cortex	Cervical nerve root compression
12	13	0.977	2010	Chronic stroke	Stroke rehabilitation	Cervical nerve root compression
13	12	0.95	1992	Motor evoked-potential	Motor evoked-potential	Human motor cortex
14	11	0.991	1995	Motor cortex	Hand muscle	Cervical nerve root compression
15	9	1	1992	Human motor cortex	Intraoperative study	Human motor cortex
16	8	0.938	2001	Therapeutic application	Therapeutic application	Therapeutic application

NIBS: Non-invasive brain stimulation; LSI: Latent semantic indexing; LLR: Log-likelihood ratio; MI: mutual information

### Emerging trends

Articles exhibiting bursts of citations indicate a notable surge In research interest within the NIBS field. Table 5 enumerates the top 10 references displaying the most pronounced citation bursts from 1988 to 2022. The initial three references underscore the emerging trend of NIBS research from 1998 to 2007, while the middle three highlight the emerging trend of new research from 2005 to 2017. The last four references, from 2013 to 2022, received significant attention and were the focus of current NIBS research.

**Table 5.** Top 10 references with the strongest citation bursts

References	Year	Strength	Begin	End	1988 - 2022	Burst	Cluster ID
Ziemann U, Ann Neurol (Ziemann et al. 1996)	1996	128.51	1998	2007	--------	128.51	9
Chen R, Neurology (Chen et al. 1997)	1997	145.67	1998	2007	--------	145.67	2
Wassermann E, Evoked Potential (Wassermann et al. 1998)	1998	186.22	1998	2007	--------	186.22	2
Huang Y, Neuron (Huang et al. 2005)	2005	201.31	2005	2017	--------	201.31	6
Nitsche M, Brain Stimul (Nitsche et al. 2008)	2008	191.96	2008	2017	--------	191.96	10
Rossi S, Clin Neurophysiol (Rossi et al. 2009)	2009	388.36	2009	2017	--------	388.36	11
Stagg C, Neuroscientist (Stagg et al. 2011)	2011	141.33	2013	2022	--------	141.33	1
Lefaucheur J, Clin Neurophysiol (Lefaucheur et al. 2014)	2014	187.99	2014	2022	--------	187.99	11
Rossini P, Clin Neurophysiol (Rossini et al. 2015)	2015	245.37	2015	2022	--------	245.37	11
Lefaucheur J, Clin Neurophysiol (Lefaucheur et al. 2017)	2017	147.99	2018	2022	--------	147.99	11

Ziemann *et al*. ^38^ reported TMS as an assessment tool to measure the effects of antiepileptic drugs. Chen *et at*. ^39^ hypothesized that the cortical excitability reduction induced by TMS has potential clinical applications in diseases such as epilepsy and myoclonus. Huang *et al*. ^36^ described a repetitive TMS (rTMS) method that allowed long-lasting effects on the human motor cortex since conventional TMS applications had weak effects on neuronal plasticity. Stagg *et al*. ^40^ summarized the physiological effects of tDCS and introduced the theoretical framework of how tDCS influences motor learning. On the other hand, 6 out of 10 articles with the strongest citation burst focused their research on establishing guidelines for the safe and effective application of NIBS. Initially, Wassermann *et al*. ^41^ proposed guidelines derived from the International Workshop on the Safety of Repetitive Transcranial Magnetic Stimulation. Rossi *et al*. ^34^ updated the guidelines for the safety application of TMS based on an expert consensus at the conference in Siena (Italy)- Nitsche *et al*. ^37^ and Rossini *et al.*^35^ provided information to perform safe and effective application of tDCS, but Rossini et al. ^35^ updated the guidelines for the application of tDCS and TMS in the brain, the spinal cord, and the peripheral nerves.

Finally, Lefaucheur *et al*. ^42^^,^^43^ summarized the conclusions of the European expert group on the application of rTMS and tDCS on pain and depression, respectively. We should highlight that Lefaucheur *et al*. ^43^ showed their concern about the inappropriate use of tDCS since the low cost and easy application mean this treatment can be performed by the patient at home, with the danger that excessive applications produce adverse effects on the patient.

References with elevated burst values are presented in table 5. The study with the highest ranking was conducted by Rossi *et al*. ^34^ within cluster 11, boasting a burst value of 388.36. Following closely, the second-highest- ranked study was authored by Rossini *et al*. ^35^ in cluster 11, holding a burst value of 245.37. The third-ranked study, by Huang *et al*. ^36^, was found in cluster 6 and featured a burst value of 201.31. These studies are important because they described safe application guidelines for TMS and tDCS and developed new application methods for a longer-lasting effect.

## Discussion

These results indicate that NIBS as a treatment and diagnostic tool is receiving increased attention and that more research is being conducted on non-invasive brain stimulation. This exponential growth is aligned with the general scope as shown by a search performed in Pubmed, with MeSH terms of neurology, where a similar growth was observed in the studied period. Clinical Neurophysiology is a professional journal dedicated to publishing about the pathophysiology underlying diseases of the peripheral and central nervous system of humans. The journal has been included in the Web of Science since 1999 and has accumulated 7,994 publications with 25,162 citations in 2021.

Brain Stimulation specializes in the publication of neuromodulation research and centers its scope on brain stimulation, encompassing invasive and non-invasive methodologies and technologies that modify brain function via electrical, magnetic, radio-wave, or precisely targeted pharmacological stimulation. The journal has been indexed in the Web of Science since 2008 and has amassed 2,258 publications, which received 10,760 citations in 2021.

This analysis provides highly personalized information for other researchers. Álvaro Pascual-Leone is a Spanish neurologist and professor at Harvard University (USA) who studies brain plasticity and the development of transcranial magnetic stimulation in the cognitive neuroscience and neurorehabilitation field. One of his most cited studies deals with the benefits of rapid-rate transcranial magnetic stimulation (r-TMS) in depression ^44^. J. C. Rothwell investigated the modulation of motor cortex excitability and electromyographic responses of limb muscles during electrical stimulation of the motor cortex ^45^^,^^46^.

The presence of two authors from different institutions within the same article signifies a collaborative effort, and the CiteSpace software facilitates the analysis of such collaborations through a co-occurrence frequency map. Cooperation analyses of institutions and countries could help to develop teamwork and global cooperation in NIBS. It is also helpful for researchers to make the best use of available resources to increase efficiency.

The CiteSpace software provides a map of the document co-citation network with nodes and lines representing the number of citations each study has received and the ratio of co-citations of the collected studies, respectively. The most representative study was the one by Rossi *et al*. ^47^, which noted a remarkable increase in the use of conventional TMS applications over the past few decades, the development of new types of TMS -such as repetitive TMS-, advancements in technology applied in novel device designs, and the incorporation of TMS with electroencephalography (EEG), positron emission tomography (PET), and functional magnetic resonance imaging (fMRI). This information made it possible to evaluate the adverse effects more related to TMS -such as the occurrence of seizures in a large number of subjects- which resulted in the updating of the ethical considerations and guidelines for the safe application of TMS based on the expert consensus in Siena (Italy).

Six years later, Rossini *et al*. ^35^ found recent guidelines in the literature on specific aspects of non-invasive brain stimulation, such as safety ^34^, methodology ^47^, and therapeutic applications ^42^. This finding motivated them to conduct a comprehensive and up-to-date review of the theoretical, physiological, and practical facets of non-invasive electrical and magnetic stimulation in the brain, spinal cord, nerve roots, and peripheral nerves.

Huang *et al*. ^36^ observed that it had been 30 years since the electrical stimulation effect on processes like learning and memory had been discovered, but it was weak in humans and did not last longer than 30 minutes. Thus, Huang *et al*. ^36^ described an rTMS method that achieved long-lasting effects on the motor cortex.

Nitsche *et al.*^37^ considered tDCS a promising tool to modulate cortical function by stimulation with weak direct currents, but the application protocols needed adjustments to improve the comparability of research results from different laboratories. Because of this, Nitsche *et al.*^37^ proposed guidelines for applying tDCS safely and effectively. However, they knew tDCS was a young technique and that future research would make it necessary to update these guidelines.

According to the document co-citation cluster labels, it becomes apparent that experts employ non-invasive brain stimulation for therapeutic purposes and as a diagnostic tool. Therapeutic applications focus on brain stimulation of areas such as the motor cortex to recover motor or executive functions or the prefrontal to restore memory. These applications are used in the treatment of neurological pathologies like stroke and psychiatric disorders such as depression. However, non-invasive brain stimulation has also been used as a diagnostic tool through evoked potential analysis to measure cortical excitability.

Research articles that experience citation bursts indicate a notable surge in research attention within the NIBS field. The magnitude of the burst value attributed to citations serves as a metric for gauging the novelty of the research outcomes. A citation burst indicates that a specific publication is being linked to a sudden surge in citations. Additionally, a cluster encompassing multiple nodes with robust citation bursts points out an active research area or an emerging trend ^27^.

The study limitations are attributed to the characteristics of CiteSpace, which only analyzes a single database and does not normalize citation data, probably resulting in the fusion of duplicate documents. For future research, it will be crucial to examine different databases and conduct a detailed analysis of the two main techniques: tDCS and TMS.

In conclusion, drawing from the findings in CiteSpace, we deliberated on key clustering, the established research framework, and the emerging trends from the references. Exploring these results, we identified that the main knowledge domains in NIBS research are treatments to recover neurological pathologies and psychiatric disorders. From the detected bursts of citations, we concluded that the safe application of NIBS and its effects on motor or executive functions are an emerging trend in NIBS research aligned with the growing trend in neurology. The current study employed a quantitative scientometric approach to examine the advancement of NIBS research through the analysis of published references in this domain. The outcomes will serve as a valuable resource for practitioners, enabling them to gain visual insights into the recognition patterns and emerging trends.
